# Lower Vitamin D During Acute Exacerbation Is Associated with Very Severe Chronic Obstructive Pulmonary Disease

**DOI:** 10.3390/medicina61060979

**Published:** 2025-05-26

**Authors:** Larisa Alexandra Rus, Romana Olivia Popețiu, Simona Maria Borta, Anamaria Vîlcea, Dragoș Vasile Nica, Teodor Vintilă, Stana Alina Măran, Maria Pușchiță

**Affiliations:** 1 Department of Internal Medicine, Faculty of Medicine, “Vasile Goldiș” Western University of Arad, Bulevardul Revoluției 94, 310025 Arad, Romania; popetiur@gmail.com (R.O.P.); anamariavilcea33@gmail.com (A.V.); mpuschita.mp@gmail.com (M.P.); 2 Arad County Emergency Clinical Hospital, Str. Andrényi Károly Nr. 2-4, 310037 Arad, Romania; alinamaran@gmail.com; 3 The National Institute of Research—Development for Machines and Installations Designed for Agriculture and Food Industry (INMA), Bulevardul Ion Ionescu de la Brad 6, 013813 București, Romania; nicadragos@gmail.com; 4 Research Center for Pharmaco-Toxicological Evaluations, Faculty of Pharmacy, “Victor Babes” University of Medicine and Pharmacy, 300041 Timisoara, Romania; 5 Faculty of Bioengineering of Animal Resources, University of Life Sciences “King Michael I of Romania” from Timisoara, Calea Aradului 119, 300645 Timisoara, Romania; teodorvintila@usvt.ro

**Keywords:** vitamin D, COPD, 25(OH)D, acute exacerbation, systemic inflammation, CRP, IL-6, biomarker profile, GOLD stage

## Abstract

*Background and Objectives*: Vitamin D deficiency is linked to adverse outcomes in chronic obstructive pulmonary disease (COPD). Limited data exist on how vitamin D levels vary by disease severity during acute exacerbations of COPD (AECOPDs). This study aimed to determine whether the vitamin D status during AECOPDs—alongside inflammatory and hematological biomarkers—is associated with COPD severity. *Materials and Methods*: This observational study included 105 AECOPD hospitalized patients, stratified according to GOLD stages 1–2, 3, and 4. Blood samples were collected to measure serum vitamin D—as 25-hydroxyvitamin D [25(OH)D], acute phase reactants, serum calcium, and selected hematological parameters. Inter-group differences were evaluated using Kruskal–Wallis tests, with Spearman correlations applied for intra-strata associations. ROC analysis and logistic regression assessed the discriminatory power of significant biomarkers. *Results*: C-reactive protein (CRP) and fibrinogen concentrations were elevated across all COPD stages, whereas calcium and vitamin D remained consistently below normal. Interleukin (IL)-6 and 25(OH)D levels varied significantly with COPD stage (*p* = 0.033 and *p* = 0.047, respectively), with a marked drop from GOLD stage 3 to stage 4. High-IL-6 patients revealed significantly elevated CRP (*p* = 0.045), erythrocyte sedimentation rate (ESR) (*p* = 0.032), fibrinogen (*p* = 0.011), and procalcitonin (*p* = 0.044). The strongest correlations were seen between CRP, ESR, and fibrinogen (*r_s_* ≥ 0.58, *p* ≤ 0.05), indicating a coordinated acute-phase response that weakened with advancing disease. Serum 25(OH)D was a significant independent predictor of COPD severity (AUC = 0.631, *p* = 0.048), while IL-6 had a weaker predictive value, losing significance in the combined regression model. *Conclusions*: Vitamin D deficiency is more pronounced in very severe COPD, serving as a potential clinical indicator of disease severity during exacerbation episodes.

## 1. Introduction

Chronic obstructive pulmonary disease (COPD) is a term used for a heterogeneous group of lung diseases associated with airflow limitation and chronic respiratory symptoms [1]. Tobacco is the most common driver of this respiratory disorder [2]. Despite the decline in smoking rates, global COPD prevalence has increased during recent decades [2]. Projections also forecast an increase by 25% until 2050, that is, from 480 million cases in 2020 to 592 million in 2050 [3]. This rise—anticipated to occur primarily in developing countries and women—is attributed to air pollution, occupational exposures, and global population aging [3,4]. COPD thus poses a major challenge to global healthcare systems and economic resilience [5,6], with acute exacerbations of COPD (AECOPDs) accounting for a substantial share of this burden [6]. These events are characterized by increased dyspnea, worsening cough, and increasing sputum volume or purulence [7].

Vitamin D is a class of lipophilic, structurally related compounds with key roles in the homeostasis of calcium, magnesium, and phosphate ions [8]. It plays a key role in lung development and immune defense [9]. A growing body of epidemiological and biological observations supports its role in COPD development, progression, and management. There is a positive association between low vitamin D and COPD risk [10,11,12,13,14,15]. Studies on the link between vitamin D deficiency and AECOPDs have shown mixed results. Most reported a direct association with AECOPD burden [16,17,18,19,20,21], while others—including a Cochrane review—found no relevant relationships [22,23,24]. The general consensus is that severe vitamin D deficiency may predict severe COPD exacerbations and comorbid flare-ups, with routine supplementation potentially reducing inflammation and improving symptoms and outcomes during the acute phase [25]. Blood vitamin D is lower during exacerbations, and in COPD patients compared to healthy individuals [17]. However, limited data are available regarding the association between vitamin D and GOLD stage during AECOPDs [13,16,17,18]. This is an important gap since disease severity may influence both the extent of deficiency and the potential benefits of targeted supplementation.

Systemic inflammation intensifies during acute episodes, leading to changes in several routinely measured biomarkers [26]. These alterations are often associated with adverse outcomes. For example, AECOPD patients with elevated levels of acute-phase proteins—including C-reactive protein (CRP), erythrocyte sedimentation rate (ESR), and fibrinogen—are prone to frequent exacerbation episodes and longer hospital stays [26,27,28,29,30,31,32]. Enhanced concentrations of pro-inflammatory cytokines modulating this response, e.g., Il-6 or IL-8 [33,34,35], low hemoglobin (anemia) [36], changes in white blood cell indices [37,38], and low serum calcium [39,40] are also associated with negative outcomes. Procalcitonin is primarily used as a bacterial infection marker (e.g., in pneumonia or sepsis), but its relevance in AECOPD is yet to be thoroughly examined [41]. There is, however, an indication that procalcitonin may guide antibiotic therapy and help assess exacerbation severity in AECOPD [42,43].

Our central hypothesis is that blood levels of vitamin D differ across GOLD stages during acute exacerbations, potentially reflecting the underlying disease severity and inflammatory burden. We quantified absolute leukocyte count, neutrophil percentage, CRP, ESR, fibrinogen, IL-6, calcium, and procalcitonin alongside vitamin D to capture the inflammatory status of patients during these exacerbation episodes. This approach may help clarify the relationship between systemic inflammation, vitamin D deficiency, and clinical staging. Moreover, our results may support future efforts to integrate vitamin D assessment into the routine evaluation and management of COPD.

## 2. Materials and Methods

### 2.1. Study Design

This exploratory, single-site, observational study aimed to determine whether these biomarkers—vitamin D levels and the associated inflammatory profile—measured during AECOPDs can differentiate between GOLD stages. The study population included adult patients admitted to the Arad County Emergency Clinical Hospital (SCJU Arad) for AECOPDs from July 2024 to February 2025. With 54 departments and wards, this hospital serves Arad County and nearby regions—with an estimated served population of over 400,000 people [44]. This study was conducted at the Department of Pneumology of SCJU Arad. The department has 83 beds, with 52 allocated to patients admitted to the Clinical Tuberculosis Unit [43]. The protocol was reviewed and approved by the independent ethics committees (IECs) of the Arad County Clinical Hospital (approval No. 38/15 May 2024) and the “Vasile Goldiș” Western University of Arad, Romania (approval No. 17/26 March 2024). All patients or their legal caregivers received and signed informed consent forms. Patient-identifiable data were treated with strict confidentiality and in full compliance with data protection regulations [45].

### 2.2. Patients and Measurements

An acute exacerbation of COPD was defined according to the 2025 GOLD report as “an acute worsening of respiratory symptoms that results in additional therapy” [46]. The study population included both known and newly diagnosed cases of COPD. The presence and stage of COPD were determined by a pulmonary specialist using spirometry, based on the following criteria: (*i*) for diagnosis: FEV_1_/FVC ratio < 70%; (*ii*) for staging: GOLD stage 1 (mild COPD): FEV_1_ ≥ 80% predicted, GOLD stage 2 (moderate COPD): 50% ≤ FEV_1_ < 80% predicted, GOLD stage 3 (severe COPD): 30% ≤ FEV_1_ < 50% predicted, and GOLD stage 4 (very severe COPD): <30% predicted; (*iii*) onset after age 40; (*iv*) long-term exposure to risk factors, including tobacco smoke (active and passive), air pollution, and occupational exposure to dust or fumes; (*v*) history or physical evidence of dyspnea, cough, sputum, and wheezing; (*vi*) persistent, progressively worsening symptoms despite treatment; (*vii*) impaired lung function between symptoms; (*viii*) limited response to rapid-acting bronchodilator therapy; and (*ix*) marked hyperinflation or other significant abnormalities on chest radiography [1]. The diagnosis of newly identified COPD was based on spirometric measurements conducted during the stable phase of the disease, after the patient had recovered from the acute exacerbation [46]. The spirometric reference values used to estimate FEV_1_% predicted were based on the European Respiratory Society/Global Lung Function Initiative (ERS/GLI) 2012 reference equations [47].

Patients with GOLD stage 1 or GOLD stage 2 were grouped together—herein referred to as the mild-to-moderate COPD group (GOLD stage 1–2). This approach was chosen due to several key considerations. First, GOLD 1 patients are a small proportion of most study populations due to underdiagnosis or low symptom burden [46]. Combining these patients into one group can thus improve the statistical power and reliability of sub-group analyses [48]. Second, these stages share comparable pathophysiological features (e.g., inflammatory markers and immune metrics) and symptom profiles (e.g., exertional dyspnea and intermittent cough). As a result, they are often managed using similar therapeutic approaches—typically short-acting bronchodilators and low-dose maintenance therapy as needed [7,27,28]. Third, grouping mild and moderate cases together enabled us to examine early disease patterns without over-fragmenting the data.

From an initial pool of 250 patients, 105 were selected for inclusion following matching for age, sex, and smoking status. This study considered only age, sex, and smoking status—matched across COPD severity groups—and origin area as potential confounders. Other relevant variables such as body mass index (BMI), physical activity level, comorbidities, and prior corticosteroid use were not included in the analysis due to inconsistent documentation in clinical files during the acute care setting, a high proportion of missing data, lack of standardized measurement protocols, and practical limitations such as time constraints during emergency admissions and variability in clinician reporting practices. Because this study was conducted during hospital admission for acute exacerbations, priority was given to immediate management and biomarker assessment. As a result, structured data collection on lifestyle and chronic comorbidities was limited and could not be reliably incorporated across the entire cohort.

Inclusion criteria were age 40 years and above, confirmed diagnosis of COPD with documented stage 1–4, hospital admission or emergency presentation with AECOPDs, blood samples collected within 24 h of admission, and informed consent provided by the patient or legal representative. Exclusion criteria were chronic respiratory diseases (e.g., asthma, bronchiectasis, and interstitial lung disease), active malignancy (cancer), chronic inflammatory or autoimmune diseases (e.g., rheumatoid arthritis, systemic lupus erythematosus, and inflammatory bowel disease), metabolic bone disorders and vitamin D metabolism issues (e.g., hypercalcemia and sarcoidosis), current or recent vitamin D supplementation (within the last 3 months), active infection unrelated to AECOPD (e.g., urinary tract infection, cellulitis, and sepsis of other origin), use of systemic corticosteroids for >7 days prior to admission, known chronic kidney disease (stage 3 or above), recent surgery or trauma (within 30 days), and inability or unwillingness to provide consent.

Venous blood samples were obtained from each AECOPD patient as soon as possible (maximum 24 h) after hospital admission. Blood was drawn using standard aseptic techniques and divided into appropriate collection tubes based on the required analyses. Complete blood count (CBC), including absolute leukocyte count, neutrophil percentage, and hemoglobin concentration, was conducted using EDTA-anticoagulated whole blood. Leukocyte and neutrophil analyses were performed using the SYSMEX XN-1500 analyzer (Sysmex Corporation, Kobe, Japan), based on impedance variation and laser light diffraction. We prioritized absolute lymphocyte count and neutrophil percentage because they offer key insights into inflammation and immune response, especially in conditions like infections or AECOPD. These parameters are clinically relevant, cost-effective, and form the basis for the neutrophil-to-lymphocyte ratio (NLR), a widely used prognostic marker. Other WBC components provide less specific or actionable information in most cases [27,29].

Serum C-reactive protein concentrations were measured via high-sensitivity immunoturbidimetric assay with a Cobas Pro biochemistry analyzer (F. Hoffmann-La Roche, Basel, Switzerland). Erythrocyte sedimentation rate (ESR) was assessed manually using the Westergren method on citrate-anticoagulated whole-blood samples. Fibrinogen was determined in plasma obtained by centrifugation of blood collected in sodium citrate tubes and analyzed via coagulometry using the Clauss method on a Sta Compact Max 3 analyzer (Diagnostica Stago S.A.S., Asnières sur Seine, France). IL-6 levels were quantified from serum using the electrochemiluminescence immunoassay (ECLIA) method on the COBAS e 601 module, part of the Cobas 6000 analytical system (Roche Diagnostics, Mannheim, Germany). We similarly determined calcitonin and 25-hydroxyvitamin D (25(OH)D)—the main circulating form of vitamin D in the blood and the best indicator of overall vitamin D status in the body [10,11]. Serum calcium was measured in serum using the Cobas Pro Bio platform (F. Hoffmann-La Roche AG, Basel, Switzerland). All samples were processed within 1–2 h of collection. Serum and plasma aliquots were stored at –80 °C until further analysis. All analyses were conducted in an ISO-accredited clinical laboratory according to manufacturer instructions and standard operating procedures. Both samples and standards were run in triplicate.

### 2.3. Statistical Analysis

Chi-square (χ^2^) analysis was used to assess differences in sex, origin area, and smoking status across COPD stages [49]. No post hoc or pairwise comparisons were performed, as these variables were intended for descriptive group characterization. Given the exploratory nature of this study and the limited sample size—especially in the GOLD stage 4 group—a global χ^2^ approach was considered more appropriate and statistically robust. Kruskal–Wallis (K-W) tests were applied to determine how age and biological variables differ between severity strata. For variables with statistically significant K-W results, planned pairwise comparisons using Dunn’s tests with Bonferroni correction were conducted between consecutive COPD stages, that is, mild-to-moderate COPD vs. severe COPD and severe COPD vs. very severe COPD. These planned comparisons were designed to quantify biomarker changes with increasing disease severity. This approach enabled us to focus on COPD-related trends while controlling for a non-normal data distribution [1].

To investigate the interplay between systemic inflammation and hematological status across COPD severity stages, we conducted a stratified correlational analysis of analyzed variables using Spearman’s correlations. This methodology enables us to assess the strength and direction of monotonic associations between key biomarkers within each severity group. This analysis was designed to identify stage-specific patterns of inflammatory activation and hematological dysregulation, and to reveal how these associations evolve with disease progression. The strength of Spearman’s rank correlation coefficient (ρ) was interpreted according to the following guidelines: 0.00 ≤ *r_s_* ≤ 0.19, very weak correlation; 0.20 ≤ *r_s_* ≤ 0.39, weak correlation; 0.40 ≤ *r_s_* ≤ 0.69, moderate correlation; 0.70 ≤ *r_s_* ≤ 0.89, strong correlation; and 0.90 ≤ *r_s_* ≤ 1.00, very strong correlation [1].

Separate and combined ROC (Receiver Operating Characteristic) analyses were performed to evaluate the robustness and predictive contributions of significant variables. AUC (Area Under the Curve), sensitivity, specificity, optimal thresholds, and statistical significance (via the Hanley and McNeil test) were calculated. Finally, logistic regression-based leave-one-out (LOO) analysis was used to verify the consistency of each predictor’s significance across the combined model. Dichotomization was performed based on the observed dynamics of each variable across COPD severity stages, with thresholds chosen at points where statistically significant differences were identified. By anchoring dichotomization to COPD-related variation, we ensured that the resulting groupings better captured the biological relevance of these markers in relation to disease severity and improved the interpretability of the predictive models. A *p*-value less than 0.05 was considered statistically significant [50].

## 3. Results

### 3.1. Serum Parameters by COPD Severity

Table 1 reveals the sociodemographic characteristics—including age, gender, and smoking history—in patients stratified by COPD stage. The distribution of patients based on area of origin exhibited significant variation with COPD severity (χ^2^ test, *p* = 0.033), with the prevalence of advanced COPD being higher in urban areas (Table 1). The distribution of males and females across severity strata was, however, similar (χ^2^ test, *p* = 0.941); the percentage of males rose with disease progression (Table 1). Smoking status was not significantly associated with GOLD stages in this dataset (χ^2^ test, *p* = 0.672).

Age did not differ significantly across COPD severity groups (Kruskal–Wallis test, *p* = 0.292). The median age was 67 (57–75; *n* = 40) in patients with mild-to-moderate COPD, 62 (54–73; *n* = 41) in adults with severe COPD, and 67 (58–72; *n* = 24) in subjects with severe COPD. Median and interquartile range values for each variable are shown in Table 2. All groups exhibited higher-than-normal levels of C-reactive protein and fibrinogen, but calcium and vitamin D deficiencies (Table 2). The other investigated variables remained within normal reference ranges (Table 2).

Most variables showed no significant inter-strata differences (Table 2), excepting IL-6 and 25(OH)D (Table 2). Interestingly, the dynamics of these markers were similar. No significant changes in IL-6 cand 25(OH)D were seen in mild-to-moderate COPD vs. severe COPD (Dunn’s tests, *p* ≥ 0.877). However, the measured values decreased significantly in very severe compared to severe disease (Dunn’s test, *p* ≤ 0.044). IL-6 and 25(OH)D levels during AECOPDs hence most effectively differentiate very severe COPD (GOLD stage 4) from less advanced disease (GOLD stages 1–3).

### 3.2. Correlational Patterns by COPD Severity

**Figure 1:**
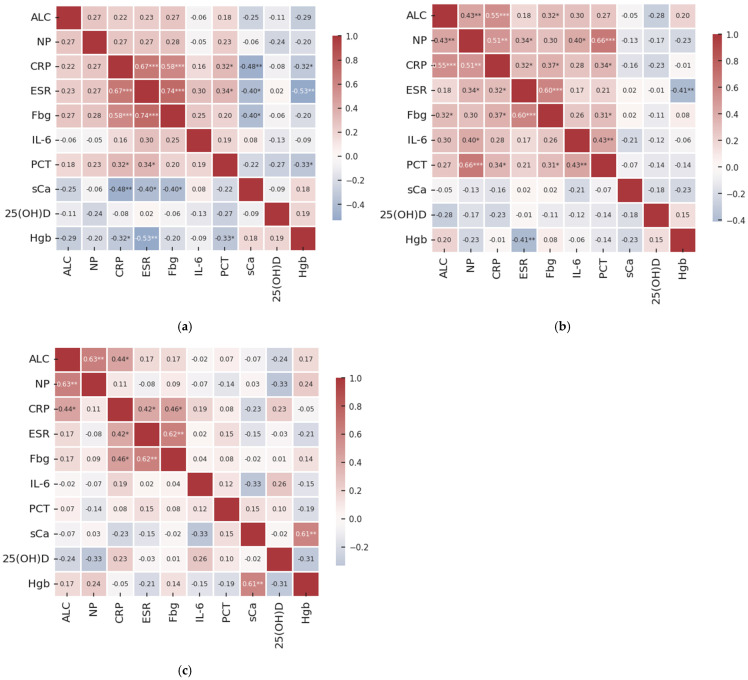
Heatmap of Spearman’s rank correlation coefficient between analyzed variables for (**a**) mild-to-moderate COPD, GOLD stage 1–2; (**b**) advanced COPD, GOLD stage 3; and (**c**) very advanced COPD, GOLD stage 4. These correlations indicate the strength of the monotonic relationship between subsequent variable pairs. Marked values (*) denote significant correlation (Spearman’s correlations, ***—*p* ≤ 0.001; **—*p* ≤ 0.01; and *—*p* ≤ 0.05).

Figure 1a–c show the Spearman correlation heatmaps depicting the relationships among key clinical variables across distinct stages of COPD severity. In patients with mild-to-moderate COPD, C-reactive protein showed moderately positive correlations with erythrocyte sedimentation ratio and fibrinogen (Figure 1a). There was also a strong association between the latter variables (Figure 1a). Procalcitonin revealed weak correlations with both C-reactive protein and erythrocyte sedimentation ratio (Figure 1a). On the other hand, serum calcium exhibited weak to moderate inverse relationships with C-reactive protein, erythrocyte sedimentation ratio, and fibrinogen (Figure 1a). Similar relationships were identified between hemoglobin levels and both C-reactive protein and erythrocyte sedimentation ratio (Figure 1a).

In severe COPD (GOLD stage 3), absolute leukocyte count revealed positive but weak-to-moderate correlations with neutrophil percentage, C-reactive protein, and fibrinogen levels (Figure 1b). Neutrophil percentage showed relationships of the same strength and direction with C-reactive protein, erythrocyte sedimentation ratio, IL-6, and procalcitonin (Figure 1b). Similar associations were observed for C-reactive protein concentrations with erythrocyte sedimentation ratio, fibrinogen, and procalcitonin (Figure 1b); for fibrinogen with erythrocyte sedimentation ratio and procalcitonin (Figure 1b); and for IL-6 with procalcitonin (Figure 1b). The only negative association was between hemoglobin and erythrocyte sedimentation ratio.

In individuals with very severe COPD (GOLD stage 4), all observed correlations were of weak to moderate strength, with most showing positive associations (Figure 1c). Leukocyte count correlated significantly with neutrophil percentage and C-reactive protein levels (Figure 1c). Erythrocyte sedimentation ratio was also associated with both C-reactive protein and fibrinogen (Figure 1c), as well as serum calcium with hemoglobin (Figure 1c). No significant associations were identified for the other variables, irrespective of COPD stage analyzed (Figure 1a–c).

### 3.3. Discriminatory Ability of IL-6 and 25(OH)D Across COPD Severity Stages

The decision to dichotomize COPD severity into non-severe and severe categories was made following initial exploratory analysis. Specifically, K-W results (see Section 3.1) suggested that the most meaningful separation occurred between very severe COPD (GOLD stage 4) and less advanced disease (GOLD stages 1–3) (see Section 3.1). Based on these findings, a binary outcome variable was constructed for logistic regression and ROC analyses, categorizing GOLD stages 1–3 as ‘less severe’ (coded as 0) and GOLD stage 4 as ‘very severe’ (coded as 1).

The outcomes of the sensitivity analysis and leave-one-out regression for IL-6 and 25(OH)D are summarized in Table 3. Both serum IL-6 and 25(OH)D level markers reached statistical significance in separating patients with advanced-stage COPD (GOLD 4) from earlier disease stages (COPD 1–3), showing moderate sensitivity but moderate-to-high specificity (Table 3). The model’s AUC improved to 0.653 when these parameters were combined, indicating potential additive value. After applying the LOO regression approach on the combined analysis of IL-6 and 25(OH)D, IL-6 lost statistical significance (Wald test, *p* = 0.481), whereas 25(OH)D remained a statistically significant independent predictor of COPD severity (Wald test, *p* = 0.047). The odds of having severe COPD decrease by approximately 6% for each 1 ng/mL increase in serum 25(OH)D while controlling for IL-6 levels.

## 4. Discussion

The interplay between vitamin D and COPD is a hot area of research. There is growing recognition of the immunomodulatory role of vitamin D, with most studies reporting its inverse association with AECOPD severity and frequency [4,16,17,18,19,20,21,22,23,24]. This relationship is evident across different GOLD stages during the stable phase of disease [14,19]. However, it remains unclear whether this association extends to the exacerbation phase. This is the first study to address this gap; we provide evidence that low vitamin D during acute exacerbations is linked to greater COPD severity. It is thus reasonable to assume that vitamin D may represent a modifiable factor in the management of high-risk patients.

The study population included a cohort of age-, sex-, and smoking status-matched individuals. This matched design minimized potential confounding factors and ensured inter-group comparability since all these variables influence systemic inflammation, vitamin D metabolism, and the natural course of COPD [2,3,5,6,9,51]. The observed associations between vitamin D levels, inflammatory markers, and COPD severity are hence likely to reflect true stage-specific biological differences, not demographic or lifestyle-related effects. The significantly higher prevalence of COPD in urban vs. rural areas is consistent with the existing literature. Available data show that urban residents are more frequently exposed to well-known risk factors for COPD progression, such as air pollutants, vehicular emissions, and industrial waste. Furthermore, higher population density, limited green space, and indoor air pollution in urban environments may contribute to more frequent exacerbations and faster disease progression [52].

IL-6 and 25(OH)D emerged as significant predictors of disease severity during these flare-ups. Serum IL-6 revealed a non-linear pattern, increasing from mild-to-moderate to severe stages before decreasing to lower levels in very severe COPD. Comparable findings have yet to be published. However, most data linking IL-6 levels and GOLD stages originate from studies on stable COPD patients and indicate that this interplay is complex. Some studies report a progressive rise in circulating IL-6 concentrations with advancing COPD severity [53,54,55]. Other investigations report no differences in IL-6 levels between GOLD stages [56,57,58]. Multiple factors may help explain these divergent findings. First, the inflammatory response differs markedly between stable and unstable disease [57] and may fluctuate depending on comorbidities, exacerbation triggers, and disease phase [59]. Second, methodological variability related to sample size, assay sensitivity, and COPD staging criteria could contribute to these divergent findings [60,61]. Third, the heterogeneity of COPD itself (e.g., emphysema-dominant vs. chronic bronchitis) might influence cytokine expression differently across patient sub-groups [3,7].

Decreased IL-6 levels in the most advanced stage of disease provide evidence for an impaired inflammatory response. Immune system exhaustion or regulatory mechanisms that suppress inflammation in severe disease may account for these results. The concept of immune system exhaustion in advanced COPD is supported by clinical observations and immunological studies. Thus, AECOPD patients display suppressed immune response, impaired regulatory mechanisms, and dysregulated immune activation vs. healthy controls and stable patients [62]. Their T-cells exhibit markers of exhaustion, such as elevated programmed death-1 (PD-1) expression, which is linked to impaired cytotoxic function [62,63].

The current understanding of vitamin D levels during AECOPDs is incomplete and evolving. It was found that most patients experiencing acute flare-ups are vitamin D-deficient, with average 25(OH)D levels below 15 ng/mL [13,16,17,18]. However, these findings primarily reflect vitamin D concentrations measured across the overall COPD population, rather than specifically comparing levels between different GOLD stages. We provide here the first quantitative data on serum 25(OH)D variations across different GOLD stages during exacerbation episodes. The measured values were consistently below normal, irrespective of COPD severity, but comparable with those reported in the aforementioned studies. Interestingly, the degree of vitamin D deficiency became clinically significant only in very severe COPD. Similar results were reported for the stable phase of disease [13,14,64]. This sharp decline in serum 25(OH)D concentrations from GOLD stage 3 to stage 4 may simply reflect physical limitations associated with very severe disease. Indeed, exercise capacity decreases with GOLD stages, leading to decreased outdoor activity, reduced sunlight exposure, and consequently, diminished endogenous vitamin D synthesis [9,65]. Advanced COPD is also associated with anorexia, weight loss, and muscle wasting [66], resulting in reduced dietary intake of vitamin D-rich foods. Other explanations involve medication used or existing comorbidities. For example, systemic corticosteroids—commonly used in advanced COPD to reduce AECOPD frequency—alter vitamin D metabolism by decreasing its absorption and increasing its degradation [7,67]. Conditions commonly associated with severe COPD, such as chronic kidney disease and heart failure [3,5], can further disrupt vitamin D metabolism and storage.

Interestingly, we identified a simultaneous and significant decline in serum IL-6 and 25(OH)D levels from severe disease (GOLD stage 3) to very severe disease (GOLD stage 4) during exacerbation events. This trend differs from the inverse relationship seen between the aforementioned variables in newly diagnosed COPD [68]. Immune exhaustion may at least partly account for these differences. Thus, systemic inflammation may cause immune exhaustion in advanced COPD—as already described in cancer [69]—leading to diminished production of pro-inflammatory cytokines like IL-6 during acute exacerbations and contrasting with earlier stages where the measured levels typically rise in response to inflammation [33,34,35]. This decline in serum IL-6 could hence indicate a compromised ability to mount an effective inflammatory response in stage 4 COPD patients. On the other hand, severe deficiency of 25(OH)D in advanced COPD may impair the synthesis and regulation of IL-6. The reduced output of vitamin D might thus contribute to the low IL-6 levels observed during exacerbations in very severe disease. There is indeed evidence that vitamin D exerts a direct regulatory effect on IL-6, downregulating its transcription in various cell types [70,71].

Inflammatory markers—C-reactive protein concentrations, erythrocyte sedimentation ratio, and fibrinogen—formed a tightly correlated inflammatory triad across all stages. These indicators were tightly linked in mild-to-moderate disease (GOLD 1–2), with inflammation negatively affecting hemoglobin and calcium levels. Inflammation can lower hemoglobin levels by disrupting iron metabolism and reducing red blood cell production—commonly seen in anemia of chronic diseases [72]. It can also reduce calcium levels by affecting metabolic regulation [73]. These changes often signal a shift from localized to systemic inflammation [74], suggesting the body’s broader response to disease progression.

Inflammation was also a major component in severe COPD (GOLD 3), but immune cell activation—reflected by absolute leukocyte counts and neutrophil percentage—and bacterial indicators—procalcitonin—gained prominence. Unresolved chronic inflammation may account for these associations [74]. In very severe disease (GOLD 4), biological relationships were less organized. While immune and inflammatory signals persisted, the system appeared exhausted or decoupled, potentially indicating disease heterogeneity or end-stage COPD physiology. The strength and number of significant biomarker correlations decreased as COPD severity increased, indicating measurement variability in advanced disease. All these findings provide evidence that immune system dysregulation most probably underlies the correlational patterns observed in this study.

We also first provide insights into the discriminatory potential of serum IL-6 and 25(OH)D levels measured during exacerbation episodes for identifying patients with very severe disease. Both biomarkers individually showed limited sensitivity, but high specificity. These indicators are hence more effective in correctly identifying patients who do not have the disease, reducing the likelihood of false-positive results. Their combination improved diagnostic performance, suggesting a potential additive effect, though this combination did not reach statistical significance in LOO regression. As a result, these biomarkers should be used in conjunction with other clinical assessments and diagnostic tools to accurately identify patients at risk.

We note that IL-6 lost statistical significance in the LOO model. In contrast, 25(OH)D remained an independent predictor of very severe COPD, with a 6% reduction in the odds of very severe disease per 1 ng/mL increase in vitamin D levels. These findings suggest that vitamin D deficiency may play a more stable and systemic role in COPD progression, independent of acute inflammatory fluctuations. We also note that the optimal 25(OH)D threshold identified here (10.3 ng/mL) aligns with available data. For example. Lokesh et al. reported that vitamin D below 18.45 ng/mL in stable patients had the highest levels of combined sensitivity and specificity for exacerbations [13].

The present study stands out through several key strengths. First, it focused on real-time 25(OH)D measurement during AECOPD. This approach provides novel insights into the dynamic relationship between inflammation and nutritional status during flare-ups, with implications for patient management and supplementation strategies. Second, our investigation merged descriptive statistics, non-parametric tests, correlation matrices, ROC analysis, and LOO regression, offering both depth and breadth of statistical analysis and capturing multi-dimensional relationships between biomarkers and disease stages. Third, this study found that both IL-6 and 25(OH)D significantly distinguish GOLD stage 4 from stages 1–3 during AECOPD, with moderate sensitivity and moderate-to-high specificity. These markers may hence be useful in risk stratification, especially in cases with limited access to spirometry. Finally, correlational analysis revealed how biological coherence weakens as COPD progresses, suggesting immune exhaustion or decoupling of inflammatory responses in GOLD 4. This nuanced analysis enhances our understanding of disease pathophysiology, offering hypotheses for future mechanistic research.

Apart from including its novel focus, robust methodology, and clinically relevant insights into biomarker dynamics during exacerbation events, this study has several limitations. Like many observatory studies, this study is single-center and lacks external validation in an independent cohort [75]. However, it lays a foundation for future multicenter work, whereas methodological transparency and rigorous inclusion/exclusion criteria increase the likelihood of reproducibility [75]. One can also argue that GOLD stage 4 was underrepresented, limiting statistical power and generalizability. We overcome this drawback by employing non-parametric tests (e.g., Kruskal–Wallis and Dunn’s) and LOO regression—both robust methods for small datasets. In addition, GOLD stage 4 COPD patients represent a naturally smaller sub-group in real-world clinical populations due to high mortality, underdiagnosis, and reduced mobility [6]. Their underrepresentation is therefore a reflection of COPD natural epidemiology [7] rather than a study design flaw. Another drawback is the loss of IL-6 significance in the multivariable model. Nonetheless, this finding provides evidence that vitamin D is a more stable predictor under multivariate conditions. The result also reflects a realistic clinical scenario where inter-marker collinearity can influence diagnostic accuracy.

Future studies should focus on confirming the causal relationship between vitamin D deficiency and COPD severity through multicenter, longitudinal studies and randomized controlled trials, especially determining the impact of supplementation during AECOPDs. In addition, upcoming research should incorporate robust multivariable models adjusting for known confounders—such as age, sex, BMI, physical activity, comorbidities, and corticosteroid use—to better isolate the independent effect of vitamin D on disease severity. Comprehensive demographic and clinical profiling—including body weight, height, smoking pack-years, spirometric data (e.g., FEV_1_%), and validated clinical scores such as mMRC (Modified Medical Research Council Dyspnea Scale), CAT (COPD Assessment Test), or SGRQ (St. George’s Respiratory Questionnaire)—will also be essential to enhance phenotype characterization and improve statistical adjustment. Further research should include detailed assessments of nutritional status and sarcopenia, such as BMI, muscle mass, and functional strength, to better understand their contribution to vitamin D deficiency and systemic inflammation in COPD. Immune profiling and cytokine analysis, including markers of T-cell exhaustion, could clarify the mechanisms behind immune dysregulation observed in GOLD stage 4. Finally, translational or in vitro studies investigating how vitamin D modulates IL-6 and other cytokines may help explain the altered inflammatory responses seen in very severe COPD.

## 5. Conclusions

This study provides the first evidence that vitamin D deficiency during AECOPDs is associated with greater disease severity, distinguishing very severe COPD (GOLD stage 4) from earlier stages. Serum 25(OH)D and IL-6 levels measured during exacerbation episodes displayed moderate sensitivity and moderate-to-high specificity in separating patients with advanced disease. Importantly, vitamin D levels remained an independent predictor of very severe COPD even after multivariable adjustment, suggesting a more stable and systemic role in disease progression compared to IL-6. The observed decline in both 25(OH)D and IL-6 concentrations in GOLD stage 4 suggest that immune exhaustion and systemic dysregulation accompany disease advancement. The weakening of biological correlations between inflammatory markers and hematologic parameters across COPD stages provides further evidence of progressive systemic deterioration in advanced disease. Our findings support the potential of real-time vitamin D assessment during AECOPD to be used for risk stratification and management of COPD patients. Moreover, they underscore the need to consider nutritional and inflammatory biomarkers as complementary tools to traditional spirometric classification, especially in acute care settings.

## Figures and Tables

**Table 1:** Sociodemographic characteristics across COPD stages.

	*n*	Age	Origin Area		Sex		Smoking Status	
			Urban	Rural	Female	Male	Non-Smoker	Smoker
Mild-to-moderate COPD	40	67 (57–75)	19 (47.5%)	21 (52.5%)	18 (45.0%)	22 (55.0%)	26 (65.0%)	14 (35.0%)
Severe COPD	41	62 (54–73)	27 (65.85%)	14 (34.15%)	17 (41.46%)	24 (58.54%)	24 (58.54%)	17 (41.46%)
Very severe COPD	24	67 (58–72)	19 (79.17%)	5 (20.83%)	10 (41.66%)	14 (58.34%)	13 (54.17%)	11 (45.83%)

The values in the third column, corresponding to the variable age, are given as medians with interquartile ranges (in parentheses). The data from the fourth column onward are shown as absolute values with the corresponding percentages in parentheses.

**Table 3:** Results of sensitivity analysis and LOO regression for IL-6 and 25(OH)D.

Sensitivity Analysis					
Variable	AUC	Optimal Threshold	Sensitivity	Specificity	*p*-Value
IL-6	0.635	2.86	0.528	0.835	0.042 *
25(OH)D	0.631	10.3	0.515	0.725	0.048 *
IL-6 + 25(OH)D	0.653	0.289	0.572	0.778	
**LOO Regression**					
	***β***	**OR**	**95% CI Lower**	**95% CI Upper**	***p*-Value**
const	−0.178	0.83	0.31	2.25	0.724
IL6	−0.006	0.99	0.97	1.01	0.481
25(OH)D	−0.061	0.94	0.88	0.99	0.047 *

IL-6, interleukin-6; 25(OH)D, 25-hydroxyvitamin D; OR, odds ratio; 95% CI, 95% confidence interval; β, coefficient beta. In sensitivity analysis, marked values (*) indicate significant differences from random classification (Hanley and McNeil test, ***—*p* ≤ 0.001; **—*p* ≤ 0.01; and *—*p* ≤ 0.05). For LOO regression, marked values (*) show significant predictors of COPD severity (Wald test, ***—*p* ≤ 0.001; **—*p* ≤ 0.01; and *—*p* ≤ 0.05).

**Table 2:** The measured values for variables analyzed across different COPD stages.

Variable	Reference Range	Mild-to-Moderate COPD	Severe COPD	Very Severe COPD	*p*-Value
ALC (×10^3^/µL)	4.00–10.00	9.70 (8.06–13.10)	10.02 (8.09–11.55)	10.60 (6.88–13.05)	0.989
NP (%)	45–80	69.50 (61.30–81.75)	74.80 (64.30–81.60)	72.20 (66.70–77.40)	0.638
CRP (mg/L)	<5.00	10.15 (3.74–24.17)	11.04 (6.01–29.16)	16.54 (4.14–57.04)	0.564
ESR (mm/h)	0–30	25 (10–30)	20 (10–30)	15 (5–35)	0.543
Fbg (mg/dL)	200–400	441 (337–598)	468 (382–538)	441 (323–508)	0.478
IL-6 (pg/mL)	0.00–7.00	6.83 (3.16–14.37)	9.23 (4.18–13.69)	3.37 (2.11–9.18)	0.033 *
PCT (ng/dL)	0.00–0.50	0.06 (0.04–0.12)	0.06 (0.04–0.11)	0.05 (0.03–0.12)	0.819
sCa (mg/dL)	8.60–10.00	7.38 (7.37–7.41)	7.39 (7.34–7.42)	7.33 (7.21–7.41)	0.297
25(OH)D (ng/mL)	30–100	16.36 (9.89–25.69)	16.43 (9.18–21.23)	10.92 (7.52–19.66)	0.047 *
Hgb (g/dL)	11.70–17.30	13.70 (12.15–15.24)	14.30 (12.90–15.70)	13.75 (12.30–14.70)	0.448

ALC, absolute leukocyte count; NP, neutrophil percentage; CRP, C-reactive protein; ESR, erythrocyte sedimentation rate; Fbg, fibrinogen; IL-6, interleukin-6; PCT, procalcitonin; sCa, serum calcium; 25(OH)D, 25-hydroxyvitamin D; Hgb, hemoglobin. The values in the second column represent the reference range based on Romanian standards. Data in the third, fourth, and fifth columns are given as medians with interquartile ranges. Marked bold values (*) in the seventh column indicate significant differences between various COPD stages (Kruskal–Wallis test, ***—*p* ≤ 0.001; **—*p* ≤ 0.01; and *—*p* ≤ 0.05).

## Data Availability

All the data generated or analyzed during this study are included in this published article.

## References

[1] Popețiu R.O., Donath-Miklos I., Borta S.M., Rus L.A., Vîlcea A., Nica D.V., Pușchiță M. (2023). Serum YKL-40 levels, leukocyte profiles, and acute exacerbations of advanced COPD. J. Clin. Med..

[2] Wheaton A.G., Liu Y., Croft J.B., VanFrank B., Croxton T.L., Punturieri A., Postow L., Greenlund K.J. (2019). Chronic obstructive pulmonary disease and smoking status—United States, 2017. Morb. Mortal. Wkly. Rep..

[3] Boers E., Barrett M., Su J.G., Benjafield A.V., Sinha S., Kaye L., Malhotra A. (2023). Global burden of chronic obstructive pulmonary disease through 2050. JAMA Netw. Open.

[4] Zheng X.-Y., Zheng Y.-J., Liao T.-T., Xu Y.-J., Liu L., Wang Y., Xiao N., Li C., He Z.-X., Tan X.-M. (2024). Effects of occupational exposure to dust, gas, vapor and fumes on chronic bronchitis and lung function. J. Thorac. Dis..

[5] Mannino D.M., Roberts M.H., Mapel D.W., Zhang Q., Lunacsek O., Grabich S., Pollack M.F. (2024). National and local direct medical cost burden of COPD in the United States. Chest.

[6] Chen S., Kuhn M., Prettner K., Yu F., Yang T., Bärnighausen T., Wang C. (2023). The global economic burden of chronic obstructive pulmonary disease for 204 countries and territories in 2020–50: A health-augmented macroeconomic modelling study. Lancet Glob. Health.

[7] Pratt A.J., Purssell A., Zhang T., Luks V.P., Bauza X., Mulpuru S., Cowan J. (2023). Complexity in clinical diagnoses of acute exacerbation of chronic obstructive pulmonary disease. BMC Pulm. Med..

[8] Ahmad R., Sarraj B., Razzaque M.S. (2025). Vitamin D and mineral ion homeostasis: Endocrine dysregulation in chronic diseases. Front. Endocrinol..

[9] Maes K., Gayan-Ramirez G., Janssens W., Feldman D., Pike J.W., Meyer M. (2024). Vitamin D and the Lung. Feldman and Pike’s Vitamin D.

[10] Lu K., Tan J.S., Li T.Q., Yuan J., Wang H., Wang W. (2023). An inverse causal association between genetically predicted vitamin D and chronic obstructive pulmonary disease risk. Front. Nutr..

[11] Mullin M.L., Milne S. (2023). Vitamin D deficiency in chronic obstructive pulmonary disease. Curr. Opin. Pulm. Med..

[12] Zhu Z., Wan X., Liu J., Zhang D., Luo P., Du W., Fan X. (2023). Vitamin D status and chronic obstructive pulmonary disease risk: A prospective UK biobank study. BMJ Open Respir. Res..

[13] Lokesh K.S., Chaya S.K., Jayaraj B.S., Praveena A.S., Krishna M., Madhivanan P., Mahesh P.A. (2021). Vitamin D deficiency is associated with chronic obstructive pulmonary disease and exacerbation of COPD. Clin. Respir. J..

[14] Zhu M., Wang T., Wang C., Ji Y. (2016). The association between vitamin D and COPD risk, severity, and exacerbation: An updated systematic review and meta-analysis. Int. J. Chronic Obstr. Pulm. Dis..

[15] Hanson C., Rutten E.P.A., Wouters E.F.M., Rennard S. (2013). Diet and vitamin D as risk factors for lung impairment and COPD. Transl. Res..

[16] Zhou L., Han C., Zhou Y. (2025). The role of severe vitamin D deficiency in predicting the risk of severe exacerbation in patients with chronic obstructive pulmonary disease. Int. J. Chronic Obstr. Pulm. Dis..

[17] Morsi M.Y.M., Shalan I.M., Sayed W.H. (2023). Study of the role of vitamin d deficiency in patients with acute exacerbation of chronic obstructive pulmonary disease. Al-Azhar Int. Med. J..

[18] Bhat M., Dar S., Waseem M., Nadeem M. (2020). Baseline vitamin d as a predictor of mortality among hospitalized patients with acute exacerbations of chronic obstructive pulmonary disease in an endemically vitamin D-deficient area in North India. Indian J. Respir. Care.

[19] Burkes R.M., Ceppe A.S., Doerschuk C.M., Couper D., Hoffman E.A., Comellas A.P., Viviano L. (2020). Associations among 25-Hydroxyvitamin d levels, lung function, and exacerbation outcomes in COPD: An analysis of the SPIROMICS cohort. Chest.

[20] Bellocchia M., Boita M., Patrucco F., Ferrero C., Verri G., Libertucci D., Bucca C. (2015). Vitamin D deficiency and COPD exacerbations: Effect of vitamin D supplementation. Eur. Respir. J..

[21] Malinovschi A., Masoero M., Bellocchia M., Ciuffreda A., Solidoro P., Mattei A., Bucca C. (2014). Severe vitamin D Deficiency is associated with frequent exacerbations and hospitalization in COPD patients. Respir. Res..

[22] Williamson A., Martineau A.R., Jolliffe D., Sheikh A., Janssens W., Sluyter J., Griffiths C.J. (2024). Vitamin D for the management of chronic obstructive pulmonary disease. Cochrane Database Syst. Rev..

[23] Martineau A.R., Jolliffe D.A., Hooper R.L., Greenberg L., Aloia J.F., Bergman P., Camargo C.A. (2017). Vitamin D supplementation to prevent acute respiratory tract infections: Systematic review and meta-analysis of individual participant data. BMJ.

[24] Kunisaki K.M., Niewoehner D.E., Connett J.E. (2012). Vitamin D Levels and risk of acute exacerbations of chronic obstructive pulmonary disease: A prospective cohort study. Am. J. Respir. Crit. Care Med..

[25] Rocha L., Figueiredo B., Martins S.E. (2025). How important is Vitamin D supplementation in the prevention of exacerbations in patients with chronic obstructive pulmonary disease (COPD): An evidence-based review. Cureus.

[26] Ko F.W., Chan K.P., Hui D.S., Goddard J.R., Shaw J.G., Reid D.W., Yang I.A. (2016). Acute exacerbation of COPD. Respirology.

[27] Chen Y.W.R., Leung J.M., Sin D.D. (2016). A systematic review of diagnostic biomarkers of COPD exacerbation. PLoS ONE.

[28] Mou S., Zhang W., Deng Y., Tang Z., Jiang D. (2022). Comparison of CRP, procalcitonin, neutrophil counts, eosinophil counts, sTREM-1, and OPN between pneumonic and nonpneumonic exacerbations in COPD patients. Can. Respir. J..

[29] Ramya P.A., Mohapatra M.M., Saka V.K., Kar R., Chakkalakkoombil S.V., Vemuri M.B. (2023). Haematological and inflammatory biomarkers among stable COPD and acute exacerbations of COPD patients. Sultan Qaboos Univ. Med. J..

[30] Sun W., Cao Z., Ma Y., Wang J., Zhang L., Luo Z. (2022). Fibrinogen, a promising marker to evaluate severity and prognosis of acute exacerbation of chronic obstructive pulmonary disease: A retrospective observational study. Int. J. Chronic Obstr. Pulm. Dis..

[31] Gjerazi J., Tashi E., Tashi I., Bushati J. (2019). Seric markers and cell profile in blood and sputum in chronic obstructive pulmonary disease exacerbations (AECOPD). Int. J. Respir. Pulm. Med..

[32] Ilisie M., Davidescu L., Genda A., Ulmeanu R. (2014). Fibrinogen and CRP biomarkers in patients with exacerbation of COPD group C and D. Eur. Respir. J..

[33] Vitkina T.I., Sidletskaya K.A. (2018). The role of interleukin-6 signaling in development of systemic inflammation in chronic obstructive pulmonary disease. Bull. Physiol. Pathol. Respir..

[34] Zhang J., Bai C. (2018). The significance of serum interleukin-8 in acute exacerbations of chronic obstructive pulmonary disease. Tanaffos.

[35] Huang A.X., Lu L.W., Liu W.J., Huang M. (2016). Plasma inflammatory cytokine IL-4, IL-8, IL-10, and TNF-α levels correlate with pulmonary function in patients with asthma-chronic obstructive pulmonary disease overlap syndrome. Med. Sci. Monit..

[36] Balasubramanian A., Henderson R.J., Putcha N., Fawzy A., Raju S., Hansel N.N., MacIntyre N.R., Jensen R.L., Kinney G.L., Stringer W.W. (2021). Haemoglobin as a biomarker for clinical outcomes in chronic obstructive pulmonary disease. ERJ Open Res..

[37] Luo Z., Zhang W., Chen L., Xu N. (2021). Prognostic value of neutrophil: Lymphocyte and platelet: Lymphocyte ratios for 28-day mortality of patients with AECOPD. Int. J. Gen. Med..

[38] Yoon E.C., Koo S.-M., Park H.Y., Kim H.C., Kim W.J., Kim K.U., Jung K.-S., Yoo K.H., Yoon H.K., Yoon H.-Y. (2024). Predictive role of white blood cell differential count for the development of acute exacerbation in Korean chronic obstructive pulmonary disease. Int. J. Chronic Obstr. Pulm. Dis..

[39] Deep A., Behera P.R., Subhankar S., Rajendran A., Rao C.M. (2023). Serum electrolytes in patients presenting with acute exacerbation of chronic obstructive pulmonary disease (COPD) and their comparison with stable COPD Patients. Cureus.

[40] Qin J., Deng X., Wei A., Qin Y., Wu Y., Liao L., Lin F. (2019). Correlation between hypocalcemia and acute exacerbation of chronic obstructive pulmonary disease in the elderly. Postgrad. Med..

[41] Pantzaris N.D., Spilioti D.X., Psaromyalou A., Koniari I., Velissaris D. (2018). The use of serum procalcitonin as a diagnostic and prognostic biomarker in chronic obstructive pulmonary disease exacerbations: A literature review update. J. Clin. Med. Res..

[42] Qiyuan P., Changyang L., Gaigai L., Ju Q., Xun Z. (2024). Prognostic value of procalcitonin in acute exacerbation of chronic obstructive pulmonary disease: A systematic review and meta-analysis. PLoS ONE.

[43] Jafari Nejad S.H., Behzadi A., Shafiepour M., Dalfardi B., Langari A.A., Ahmadipour H., Fekri M.S. (2023). Comparison of serum procalcitonin levels between patients with acute exacerbation of chronic obstructive pulmonary disease (AECOPD) and patients with chronic obstructive pulmonary disease. Curr. Respir. Med. Rev..

[44] Spitalului Clinic Județean de Urgență Arad. https://www.scjarad.ro/pneumologie-i/.

[45] Grelus A., Nica D.V., Miklos I., Belengeanu V., Ioiart I., Popescu C. (2017). Clinical significance of measuring global hydroxymethylation of white blood cell DNA in prostate cancer: Comparison to psa in a pilot exploratory study. Int. J. Mol. Sci..

[46] Global Initiative for Chronic Obstructive Lung Disease (2023). Global Strategy for the Diagnosis, Management, and Prevention of Chronic Obstructive Pulmonary Disease. https://goldcopd.org/wp-content/uploads/2023/03/GOLD-2023-ver-1.3-17Feb2023_WMV.pdf.

[47] Cooper B.G., Stocks J., Hall G.L., Culver B., Steenbruggen I., Carter K.W., Thompson B.R., Graham B.L., Miller M.R., Ruppel G. (2017). The Global Lung Function Initiative (GLI) network: Bringing the world’s respiratory reference values together. Breathe.

[48] Song F., Bachmann M.O. (2016). Cumulative subgroup analysis to reduce waste in clinical research for individualised medicine. BMC Med..

[49] Georgescu M., Drăghici G.A., Oancea E.F., Dehelean C.A., Şoica C., Vlăduţ N.V., Nica D.V. (2021). Effects of cadmium sulfate on the brown garden snail *Cornu aspersum*: Implications for DNA Methylation. Toxics.

[50] Drăghici G.A., Dehelean C., Pinzaru I., Bordean D.M., Borozan A., Tsatsakis A.M., Nica D. (2019). Soil copper uptake by land snails: A semi-field experiment with juvenile *Cantareus aspersus* snails. Environ. Toxicol. Pharmacol..

[51] Yuan L., Ni J. (2022). The association between tobacco smoke exposure and vitamin D levels among US general population, 2001–2014: Temporal variation and inequalities in population susceptibility. Environ. Sci. Pollut. Res..

[52] Sicard P., Agathokleous E., Anenberg S.C., De Marco A., Paoletti E., Calatayud V. (2023). Trends in urban air pollution over the last two decades: A global perspective. Sci. Total Environ..

[53] Nieri D., Daniele M., Lombardi S., Bazzan E., Santerini S., De Cusatis G., Neri T. (2021). Circulating extracellular vesicles are associated with disease severity and interleukin-6 levels in COPD: A pilot study. J. Clin. Med..

[54] Yadav R.S., Kant S., Tripathi P.M., Pathak A.K., Mahdi A.A. (2022). Transcript levels of COX-2, TNF-α, IL-6 and IL-10 in chronic obstructive pulmonary disease: An association with smoking and severity. Res. J. Biotechnol..

[55] Singh S., Verma S.K., Kumar S., Ahmad M.K., Nischal A., Singh S.K., Dixit R.K. (2018). Correlation of severity of chronic obstructive pulmonary disease with potential biomarkers. Immunol. Lett..

[56] Abd Elnaby E.A., Abd Elnaiem S.S., Mostafa A.I., Sabry D., Alnaggar A.R.I., Haswa M.K. (2019). Assessment of serum interleukin 6 level in patients with chronic obstructive pulmonary disease: Is it related to disease severity?. Egypt. J. Bronchol..

[57] Wei J., Xiong X.F., Lin Y.H., Zheng B.X., Cheng D.Y. (2015). Association between serum interleukin-6 concentrations and chronic obstructive pulmonary disease: A systematic review and meta-analysis. PeerJ.

[58] de Moraes M.R., da Costa A.C., Corrêa K.D.S., Junqueira-Kipnis A.P., Rabahi M.F. (2014). Interleukin-6 and interleukin-8 blood levels’ poor association with the severity and clinical profile of ex-smokers with COPD. Int. J. Chronic Obstr. Pulm. Dis..

[59] Harvanová G., Duranková S. (2025). Inflammatory process: Factors inducing inflammation, forms and manifestations of inflammation, immunological significance of the inflammatory reaction. Alergol. Pol.-Pol. J. Allergol..

[60] Mulvanny A., Pattwell C., Beech A., Southworth T., Singh D. (2022). Validation of sputum biomarker immunoassays and cytokine expression profiles in COPD. Biomedicines.

[61] Gamble E., Qiu Y., Wang D., Zhu J., Vignola A.M., Kroegel C., Jeffery P.K. (2006). Variability of bronchial inflammation in chronic obstructive pulmonary disease: Implications for study design. Eur. Respir. J..

[62] Xiong X.F., Zhu M., Wu H.X., Wu Z.H., Fan L.L., Cheng D.Y. (2025). T-cell immune status in patients with acute exacerbation of chronic obstructive pulmonary disease: A case-control study. Front. Med..

[63] Wilkinson T.M. (2017). Immune Checkpoints in Chronic Obstructive Pulmonary Disease. Eur. Respir. Rev..

[64] Zheng Z., Zhu B., Zhu B., Xiao C. (2015). Vitamin D deficiency is associated with the severity of COPD: A systematic review and meta-analysis. Int. J. Chronic Obstr. Pulm. Dis..

[65] Blondeel A., Hermans F., Breuls S., Wuyts M., De Maeyer N., Verniest T., Demeyer H. (2023). The Association of weather conditions with day-to-day variability in physical activity in patients with COPD. ERJ Open Res..

[66] De Brandt J., Beijers R.J., Chiles J., Maddocks M., McDonald M.L.N., Schols A.M., Nyberg A. (2022). Update on the etiology, assessment, and management of COPD cachexia: Considerations for the clinician. Int. J. Chronic Obstr. Pulm. Dis..

[67] Mjid M., Zargouni A., Hedhli A., Euchi K., Ouahchi Y., Cheikhrouhou S., Dhahri B. (2023). Does corticosteroid intake increase the risk of vitamin D deficiency in Tunisia’s COPD patients?. Afr. Health Sci..

[68] Fu L., Fei J., Tan Z.X., Chen Y.H., Hu B., Xiang H.X., Xu D.X. (2021). Low vitamin D status is associated with inflammation in patients with chronic obstructive pulmonary disease. J. Immunol..

[69] Fang L., Liu K., Liu C., Wang X., Ma W., Xu W., Sun C. (2022). Tumor Accomplice: T cell exhaustion induced by chronic inflammation. Front. Immunol..

[70] Nikkholgh A., Tavakoli F., Alborzi N., Araste F. (2023). Vitamin D attenuates cardiac hypertrophy in rats through mRNA Regulation of interleukin-6 and its receptor. Res. Cardiovasc. Med..

[71] Xu L., Lee M., Jeyabalan A., Roberts J.M. (2013). The Relationship of hypovitaminosis D and IL-6 in preeclampsia. Am. J. Obstet. Gynecol..

[72] Lanser L., Fuchs D., Kurz K., Weiss G. (2021). Physiology and inflammation driven pathophysiology of iron homeostasis—Mechanistic insights into anemia of inflammation and its treatment. Nutrients.

[73] Iamartino L., Brandi M.L. (2022). The calcium-sensing receptor in inflammation: Recent updates. Front. Physiol..

[74] Qi Y., Yan Y., Tang D., Han J., Zhu X., Cui M., Fan F. (2024). Inflammatory and immune mechanisms in COPD: Current status and therapeutic prospects. J. Inflamm. Res..

[75] Overhage J.M., Overhage L.M. (2013). Sensible Use of Observational Clinical Data. Stat. Methods Med. Res..

